# Quantification of cyanobacterial cells via a novel imaging-driven technique with an integrated fluorescence signature

**DOI:** 10.1038/s41598-018-27406-0

**Published:** 2018-06-13

**Authors:** Chao Jin, Maria M. F. Mesquita, Jason L. Deglint, Monica B. Emelko, Alexander Wong

**Affiliations:** 1 Department of Civil and Environmental Engineering, University of Waterloo, Waterloo, Ontario N2L 3G1 Canada; 2 Department of Systems Design Engineering, University of Waterloo, Waterloo, Ontario N2L 3G1 Canada

## Abstract

A novel imaging-driven technique with an integrated fluorescence signature to enable automated enumeration of two species of cyanobacteria and an alga of somewhat similar morphology to one of the cyanobacteria is presented to demonstrate proof-of-concept that high accuracy, imaging-based, rapid water quality analysis can be with conventional equipment available in typical water quality laboratories-this is not currently available. The results presented herein demonstrate that the developed method identifies and enumerates cyanobacterial cells at a level equivalent to or better than that achieved using standard manual microscopic enumeration techniques, but in less time, and requiring significantly fewer resources. When compared with indirect measurement methods, the proposed method provides better accuracy at both low and high cell concentrations. It extends the detection range for cell enumeration while maintaining accuracy and increasing enumeration speed. The developed method not only accurately estimates cell concentrations, but it also reliably distinguishes between cells of *Anabaena flos-aquae*, *Microcystis aeruginosa*, and *Ankistrodesmus* in mixed cultures by taking advantage of additional contrast between the target cell and complex background gained under fluorescent light. Thus, the proposed image-driven approach offers promise as a robust and cost-effective tool for identifying and enumerating microscopic cells based on their unique morphological features.

## Introduction

Cyanobacteria, commonly known as blue-green algae, are a group of diverse photosynthetic bacteria that occupy a broad range of aquatic and terrestrial habitats, contributing to global biodiversity and bio-geochemical cycles^1,2^. In recent decades, anthropogenic activities and climate change have contributed to increases in cyanobacteria occurrence in surface waters, often leading to excessive growth and/or potentially harmful algae blooms (HABs)^3–5^. Although a complex interaction of environmental factors has been shown to contribute to these blooms, the exact triggers that determine their occurrence are still poorly understood. During bloom events, some cyanobacteria species may produce taste/odor compounds (e.g., geosmin, 2-methylisoborneol) and toxic metabolites (cyanotoxins), which can threaten human and animal health, and disrupt the balance of aquatic ecosystems^6–8^. As a result, many jurisdictions have introduced specific water quality regulations to protect public health and safety^8–10^.

Currently, drinking water quality guidelines related to cyanobacteria are based on maximum acceptable concentrations of toxins (e.g., microcystin-LR) in treated water (e.g., 1.0 µg/L proposed in WHO, 1999) or elevated levels of cyanobacterial cells (e.g., ≥100,000 cells/ml) in water supplies^7^. In practice, routine quantitative cyanotoxin monitoring in water is both expensive and challenging because it is time consuming and requires sophisticated equipment and methodologies, as well as substantial technical expertise^11^. Direct microscopic enumeration of cyanobacterial cells in water offers a simpler and more cost effective alternative to these approaches; as a result, it has become common practice even though it is limited by relatively high detection limits (e.g., ~10^5^ cells/ml when using a typical hemacyotmeter) and the time required for identification, confirmation, and enumeration. The World Health Organization (WHO) released guidance values for recreational exposure based on cyanobacterial cell counts and chlorophyll-a concentrations^12^; three alert levels of severity and probability of health effects based on cell concentrations (i.e., low up ≤2*10^4^ cells/ml, moderate ≤10^5^ cells/ml but ≤2*10^4^ cells/ml, and high >10^5^ cells/ml) were defined. In Canada, the recommended guideline for recreational water use is <10^5^ cells/ml of total cyanobacteria^8,13^. Recreational water guidelines in Australia and New Zealand specify that these waters should not contain >5 × 10^4^ cells/ml (or biovolume equivalents of >4 mm^3^/L for the combined total of all cyanobacteria where a known toxin producer is dominant; or >10 mm^3^/L for the total biovolume of all cyanobacterial material where known toxins are not present or cyanobacterial scums consistently present)^9,10,14^. In the United States, several states have implemented bloom response guidelines that also include cell counts in the event of a significant cyanobacterial bloom^15^. Thus, accurate identification and measurement of cyanobacteria concentrations or biovolume estimation are important for watershed management, potable water production, recreational use, and water re-use.

Cost effective, fast, and reliable cyanobacterial cell identification and enumeration methods are thus much-needed, essential components of water quality monitoring programs. A number of direct and indirect analytical methods are currently available for these analyses (Table S-1). Despite its limitations, direct microscopic enumeration using a hemacytometer is still the most commonly used cyanobacterial cell identification and enumeration method. When applied to natural samples it presents challenges associated with weak contrast of cells against the background, high species diversity, variable morphology of individual cells, and complexity of cell aggregates or units (i.e. colonies, entangled filaments etc.). Typically, unicellular genera are directly counted and reported as cells/ml while colonial species are disintegrated before enumeration^16^ and filamentous genera are counted as number of filaments, cells per filament or total filament length per mL^7^. Notably direct enumeration using a hemacytometer is generally inaccurate at lower cell concentrations, though it depends on the specific hemacytometer and cell species being enumerated, often requiring analysts to view more area over multiple chambers to get reliable results^17^. When individual cell counts are not possible, the geometric volume of individual units (i.e. colonies/filaments) can be estimated by determining the average cell volume for each species or unit counted and then multiplying this value by the cell number present in the sample. Average volumes are determined by assuming idealized geometric bodies for each species (e.g. spheres for *Microcystis* cells, cylinders for filaments), measuring the relevant geometric dimensions of 10 to 30 cells (depending upon variability) of each species, and calculating the corresponding mean volume of the respective geometric body (WHO 1999)^6^.

Indirect quantification methods such as flow cytometry^18^, antibody-mediated immunofluorescence microscopy assays^19^, PCR-fluorescent fragment detection^20^, qPCR^21,22^, molecular probes using sandwich hybridization (SHA)^23^, and *in-situ* fluorescence^24^ also have been developed to estimate cyanobacterial cell concentrations in water. However they frequently require expensive equipment not found in typical water quality laboratories. As well, flow cytometry is not always accurate/specific enough to distinguish cells from other particulate matter in the water matrix; molecular biology-based methods are costly and non-specific, while non-specific binding may occur with polyclonal techniques resulting in overestimation of cell concentration^25^. Indirect methods based on the fluorescence of photosynthetic pigments produced by the cyanobacteria (especially chlorophyll-a) are not specific to the target cells and may thus, also result in overestimation^26–28^. In recent years, lab-on-chip technologies integrating cell preparations (i.e. cell sorting, trapping etc.), signal processing, and data analysis have become more common^29,30^. However, most of these technologies were originally developed in clean environments and are not ready to be implemented in routine water monitoring due to their insufficiency in dealing with real, complex water matrices and the occurrence of positive/negative error associated with automated sensing^31^.

In contrast to the aforementioned methods, imaging-based enumeration methods are more promising for rapid and low-cost water quality monitoring of cyanobacteria due to their several important advantages. Specifically, these methods are relatively more: 1) cost effective and rapid, 2) specific because they can detect and differentiate target cells based on size, shape, and color, 3) readily integrated into current monitoring protocols, 4) automated, 5) customizable/adaptable, and 6) flexible for development of quality control and assurance protocols. Nevertheless, imaging–based methods still present technical challenges that need to be overcome before automated cell enumeration can be achieved and routinely used for accurate and reliable water quality analysis. One challenge is associated with the low contrast between cyanobacterial cells (mostly transparent and lightly colored) and the background water matrix, which makes it difficult to capture the target cells in clear images. Differential interference contrast, cell staining and epifluorescence have been used to increase contrast and facilitate the enumeration process. Another challenge is that cyanobacteria often occur as multicellular structures of complex morphology, in which individual cells are difficult to identify and enumerate^32^. For example, in filamentous genera cells are often not in the same microscopic focal plane (e.g., *Anabaena sp*. filaments can reach 50 μm to >2000 μm in length), which may result in significant underestimation of cell number or bio-volume. Due to manufacturer-associated differences in microscope and camera characteristics, intensive manual tuning is often required, making it difficult to develop universally applicable, automated methods. Finally, a commonly used approach in image processing is thresholding, in which “a binary image whose one state will indicate the foreground objects (i.e. printed text, a legend, a target defective particle of a material, etc.) [is used] while the complementary state will correspond to the background”^33^. Notably, factors such as (1) non-stationary and correlated noise, (2) ambient illumination, (3) complexity within the objectives associated with gray levels, (4) inadequate contrast and (5) un-proportional objectives can rendering thresholding methods ineffective for the identification and enumeration of cyanobacterial cells in natural waters^34^. Other automatic segmentation methods such as active contours^35^ and level set techniques^33,36^ are not feasible for use in microbiological sample enumeration because they cannot resolve the challenges associated with background illumination and objective aggregation^37^. Preliminary work has explored the possibility of using a statistical image based approach to enumerate cyanobacteria concentrations in lab-culture environments with reasonable success^38^; however, those results have not been rigorously tested in real water matrices, especially those containing other fluorescing particulate matter. Mechanistic investigations of contrast enhancement at different wavelengths have not been conducted either, preventing application of these techniques in water monitoring.

This work was conducted to demonstrate that contemporary image analysis and processing technology has advanced to a level that enables good identification and enumeration of algae in real (untreated) water matrices in a manner that is (1) rapid and automated (and enables for analyst confirmation) without requiring additional expensive equipment (e.g., flow cytometry) beyond that which is commonly available for microbiological analyses in typical water quality laboratories and (2) at least as accurate as currently available methods (e.g., hemacytometer, fluorometry), but with greater sensitivity (i.e., lower detection limit). This work is intended as a proof-of-concept investigation that demonstrates that image processing algorithms that utilize fluorescence enhancement and an adequate extent of morphological characterization can distinguish both distinctly different and similar microbe morphologies; thus, these technologies should be more extensively studied to develop reasonably low cost, rapid screening tools for evaluating water quality and public health risk.

In this study, a two-phase model-driven method of automated enumeration was developed to quantify the cell concentration (i.e. cells/ml) of two representative species of freshwater cyanobacteria (*Microcystis aeruginosa* and *Anabaena flos-aquae*). Fluorescent light was used to excite the natural photosynthetic pigments in the cells for contrast enhancement. A probabilistic unsupervised classification approach was developed to distinguish the target cells from their surrounding background. In Phase 1, quantitative information associated with individual cells (e.g., dimensions and morphology) was analyzed and used for model calibration. In Phase 2, the quantitative information obtained in Phase 1 was used to separate the target cells from the background matrix and estimate their concentration. For method validation purposes, enumeration results obtained using the newly proposed automated method consisting of total counts of fluorescent cells in images of a filled hemocytometer; they were compared to those obtained by: a) direct standard enumeration using bright field microscopy and the same hemocytometer (AWWA 10200 F)^11^, and b) indirect measurement using fluorometric probes (EXO2 Multiparameter Sonde, Total Algae PC smart Sensor, YSI Yellow Springs, Ohio USA). Laboratory cultured cyanobacterial cells suspended in an aqueous isotonic phosphate buffered saline solution (PBS) and in a natural lake water matrix of moderate turbidity (4.7 ± 0.6NTU) were used for method comparison and validation purposes only. Further testing was then conducted to differentiate between cells of relatively similar morphologies. Specifically, a mixed suspension of *Microcystis, Anabaena, and Ankistrodesmus* was utilized and several morphological attributes were evaluated for differentiation, including eccentricity, compactness, convex area, solidity, extent and perimeter were selected to recognize the microscopic cells of interest.

## Materials and Methods

### Cyanobacteria, algae, and growth conditions

Two cyanobacteria cultures (*Microcystis aeruginosa* and *Anabaena flos-aquae*) were originally obtained from the Canadian Phycological Culture Centre (CPCC) at University of Waterloo and cultured in sterile BG-11 medium^39^ in glass flasks. After autoclaving and cooling, the pH of the medium was adjusted to 7.4 and 1 mL of a filter sterilized vitamin solution containing trace amounts of vitamin B12, biotin and thiamine was added^40^. The cyanobacterial cells were grown in a Percival growth cabinet (John’s Scientific Inc., Canada) at a temperature of 22 ± 1 °C and exposed to a full spectrum of white light (~1200 lumens) for 12 hours followed by darkness for 12 hours for 10 to 15 days before image acquisition.

*Microcystis aeruginosa* and *Anabaena flos-aquae* were selected for use in the present study because they are two of the most common toxigenic cyanobacteria species present in freshwater globally and both can produce harmful cyanotoxins during bloom conditions^4^. *Microcystis aeruginosa* is representative of unicellular, spherical and relatively uniform cyanobacterial cells because when grown under nutrient rich conditions (i.e., in laboratory cultures) it produces individual rather than aggregated cells, allowing for easier and more reliable enumeration, thereby making it a good candidate for method validation. *Anabaena flos-aquae* was chosen to represent filamentous cyanobacteria, which are particularly difficult to enumerate using traditional methods^11^.

The green algae *Ankistrodesmus* was also obtained from CPCC. These cells are long and needle- or spindle-shaped, or sometimes curved or slightly crescent-shaped. While not identical, they can be similar in morphology to the filamentous cyanobacteria *Anabena*; thus, their use enabled a preliminary proof-of-concept demonstration of cell differentiation using the imaging-based method presented herein.

### Cyanobacteria microscopic enumeration

Standard cyanobacterial cell enumeration was performed under bright field using an AXIOSKOP 2 Plus microscope (Zeiss, Germany) at 400 × total magnification and a Bright-Line Hausser hemocytometer-3100 (Horsham, PA) with two separate 10 µL compartments with counting grids. Counting was performed in triplicate in 1 mm^3^ following the instrument technical instructions provided by the manufacturer and recommendations by AWWA^17^. Enumeration by the newly proposed automated method was performed in images of the fluorescing cells obtained using the same microscope and hemocytometer. It should be noted that hemacytometer detection limits are typically ~10^5^ cells/ml because the statistics of discrete counts conform to the Poisson distribution^41^; thus, estimates of cell concentrations that are based on low cell counts and obtained with hemocytometers are imprecise^42^. Accordingly, the estimated values less than 10^5^ cells/ml that were obtained with the hemacytometer are included in the figures herein only for the purpose of visualization.

### Image acquisition

**Figure 1:**
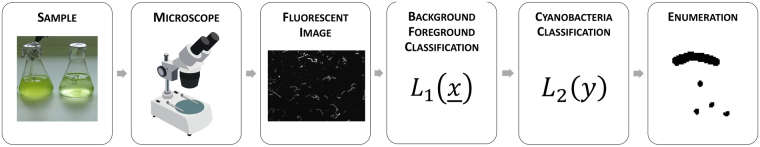
Schematic of overall process of image acquisition and analysis.

An overall schematic of the enumeration process and numerical method is provided in Fig. 1.

Fluorescent signal acquisitions were collected during the imaging process using the Axioscope2 Plus Zeiss microscope, a 10X objective, and a CCD camera (QImaging Retiga EXi Mono 12 bit, 1600 × 1200 pixels, Fast 1394). Each sample was exposed to various fluorescent light wavelengths (365, 450–490 and 546 nm wavelength) via FITC/AO filter cubes (Zeiss filter sets 2, 9 and 15). In Phase 1, quantitative information related to unit-cell morphology, size, and distribution was analyzed. Cultured cells were sonicated to break aggregates and filaments into shorter segments or isolated single cells. Different sonication periods (0.5, 1, 2, 5 and 10 minutes) were evaluated. Sonicated suspensions were aseptically filtered through a 4.5 µm sterile filter membrane placed on top of an 8 μm supporting membrane (Whatman Inc., Clifton NJ). The purpose of this step was to remove remaining long filaments and/or cell aggregates from suspension by size exclusion, therefore ensuring that only well isolated single cell dimensions were included in the model calibration. The projected area per unit cell was calculated based on the active contour generated from the Maximum likelihood (ML) classifier introduced above. Five duplicate images were collected per sample.

### Image processing

Given an image acquisition, a binary classifier was used to classify each pixel in the image as either part of the background *C*_*b*_ or as part of the foreground *C*_*f*_. The binary classifier L_1_ can be defined as

1 $${L}_{1}(\mathop{x}\limits_{\_})=\{\begin{array}{cc}{C}_{f} & f(\mathop{x}\limits_{\_})\ge \theta \\ {C}_{b} & {\rm{o}}{\rm{t}}{\rm{h}}{\rm{e}}{\rm{r}}{\rm{w}}{\rm{i}}{\rm{s}}{\rm{e}}\end{array}$$

where *θ* is the decision boundary between the two classes, and *f*($\mathop{x}\limits_{\_}$) is the pixel intensity at $\mathop{x}\limits_{\_}$. To learn this classifier, the pixels corresponding to a subset of the image were used to learn the decision boundary *θ* in Eq. (above equation) by maximizing the between-class variance in the training set, as originally proposed by Otsu *et al*.^43^. The advantage of this approach to learning the binary classifier is that no underlying Gaussian assumption is made about the probability density of each class, which is required when using other closed-form solutions such as the Bayes’ decision rule^44^.

Therefore, given the label field *L*_1_ produced using the binary classifier, connected component analysis was then performed to produce a set of connected components. Given the set of connected components, an additional classification was performed on each of the connected components, *y*, to determine whether they were part of the *Anabaena* class *C*_*A*_, or part of the *Microcystis* class *C*_*M*_, or part of neither class *C*_*N*_. This was accomplished by characterizing each connected component using two quantitative morphological features of each component. These were the eccentricity (*ε*), and the compactness, (*c*), as well as the area metric (*a*), which is the total area at a given connected component. Therefore an additional classifier *L*_2_ was defined as:

2 $${L}_{2}(y)=\{\begin{array}{cc}{C}_{A} & (\varepsilon (y)\ge \omega )\wedge (c(y)\le \tau )\wedge (a(y)\ge {\psi }_{AL})\wedge (a(y)\le {\psi }_{AU})\\ {C}_{M} & (\varepsilon (y) < \omega )\wedge (c(y) > \tau )\wedge (a(y)\ge {\psi }_{ML})\wedge (a(y)\le {\psi }_{MU})\\ {C}_{N} & {\rm{o}}{\rm{t}}{\rm{h}}{\rm{e}}{\rm{r}}{\rm{w}}{\rm{i}}{\rm{s}}{\rm{e}}\end{array}$$

where *ε*(*y*), *c*(*y*) and *a*(*y*) are the eccentricity, compactness and area at connected component y, respectively. In addition, *ω* and *τ* are the empirically derived decision boundaries for the eccentricity and compactness, respectively. Furthermore, *ψ*_*AL*_ and *ψ*_*AU*_ are the lower and upper area decision boundaries for *Anabaena*, and *ψ*_*ML*_ and *ψ*_*MU*_ are the lower and upper area decision boundaries for *Microcystis*, which were also empirically derived.

The eccentricity, *ε*, was calculated by fitting an ellipse to a foreground object, and then taking the ratio of the distance between the two foci. This metric is bounded between 0 and 1, where the eccentricity of a circle is 0 and the eccentricity of line segment is 1. Therefore, in general, *Anabaena* cells had high eccentricity opposed to *Microcystis* cells, which had low eccentricity. The compactness, *c*, is the ratio of the area of circle whose circumference is equal to the perimeter, *P*, to the actual area, *A, of* a given object. More specifically, compactness can be defined as *c* = 4*π*(*A*/*P*^2^) and is theoretically bounded between 0 and 1. The compactness of a circle is exactly 1, while more irregularly shaped objects have lower compactness. Accordingly, *Microcystis* cells had a high compactness when compared to those of *Anabaena*, which had a much lower compactness value. This is further illustrated in Table 1. Using these two classifiers in series, the *Anabaena* and *Microcystis* cells were automatically segmented into their respective classes. To enumerate the cells in the *Anabaena* class, first the total area of the cell class and then the previously calibrated pixels to area values from the model parameters were computed. In the case of *Microcystis*, the total number of foreground objects were counted to enumerate the cell class.

**Table 1:** Representative eccentricity and compactness values of sample images of *Anabaena* and *Microcystis* cells.

Species	Eccentricity (*ε*)	Compactness (*c*)
*Anabaena flos-aquae*	0.8537	0.6444
*Microcystis aeruginosa*	0.5205	0.9834

To investigate the robustness of the proposed method in distinguishing microscopic cell, pure cultures of *Ankistrodesmus, Microcystis* and *Anabaena* were first imaged using the same setup as described previously. Then, for each organism, the foreground and background were separated using the classifier presented in Equation 1. Once again, connected component analysis was performed. An additional set of four morphological features (convex area, solidity, extent and perimeter) were measured, resulting in a total of seven morphological features that could be used for cell recognition/differentiation (Table 2). Specifically, the convex area of a connected component is the smallest convex polygon that contains the entire set points in the connected component while the solidity is a measure of the ratio between the foreground pixels of a given object and the number of pixels in the convex hull. The extent is a measure of the ratio between the foreground pixels of a given object and the number of pixels in the bounding box of that object. Finally, perimeter is the length in pixels of the boundary of the object. These seven features were found for 272 images of each cell type and formed a dataset that could be used for quantitative evaluation of three different cells used. Therefore there was a total of 816 images each consisting of either a *Microcystis*, *Anabaena*, or *Ankistrodesmus* cell.

**Table 2:** Representative mean and standard deviation of selected features for differentiation between *Microcystis, Anabaena*, and *Ankistrodesmus*.

Value	*Microcystis*	*Anabaena*	*Ankistrodesmus*
Area	11.41 ± 5.05	150.04 ± 157.53	47.46 ± 31.84
Eccentricity	0.59 ± 0.22	0.90 ± 0.14	0.98 ± 0.03
Compactness	1.82 ± 0.34	0.61 ± 0.51	0.44 ± 0.30
Convex Area	11.63 ± 5.32	342.84 ± 711.74	63.29 ± 58.24
Solidity	0.99 ± 0.03	0.70 ± 0.22	0.81 ± 0.10
Extent	0.81 ± 0.11	0.43 ± 0.23	0.30 ± 0.18
Perimeter	8.94 ± 2.65	75.12 ± 69.45	40.93 ± 20.75

## Method Validation

To evaluate the accuracy of the proposed method, rigorous validation experiments were conducted. First, the two species of cyanobacteria used were mixed at different volumetric proportions (i.e. 100, 80, 60, 50, 40, 20 and 0% in total volume) to validate the effectiveness of the proposed method in distinguishing them. Results obtained using manual microscopic enumeration were compared to calculated results for validation. In addition, stock suspensions of the two cultured cyanobacteria were used to prepare serial decimal dilutions (i.e. 1, 0.5, 10, 500 and 1000 respectively). Two types of water were used for dilution. Namely: laboratory phosphate-buffered saline (PBS) solution, and untreated/raw surface water from Lake Ontario. Method validation was conducted using three cyanobacterial cell concentration evaluation methods: a) direct microscopic manual enumeration using Standard Method 10200 F (AWWA 2012), b) indirect evaluation based on chlorophyll-a and phycocyanin fluorescence measurements using a specific fluorometric probe, and c) automated image enumeration using the method developed herein. To quantify the effectiveness of the developed algorithm, the determined specificity, sensitivity and accuracy of the developed method as compared to the manual microscopic direct enumeration method were evaluated as:

3 $${\rm{S}}{\rm{p}}{\rm{e}}{\rm{c}}{\rm{i}}{\rm{f}}{\rm{i}}{\rm{c}}{\rm{i}}{\rm{t}}{\rm{y}}=\frac{TN}{TN+FP}$$

4 $${\rm{S}}{\rm{e}}{\rm{n}}{\rm{s}}{\rm{i}}{\rm{t}}{\rm{i}}{\rm{v}}{\rm{i}}{\rm{t}}{\rm{y}}=\frac{TN}{TP+FP}$$

5 $${\rm{A}}{\rm{c}}{\rm{c}}{\rm{u}}{\rm{r}}{\rm{a}}{\rm{n}}{\rm{c}}{\rm{y}}=\frac{TN+TP}{TP+TN+FP+FN}$$

6 $${\rm{F}}1=(\frac{2\ast Precision\ast Recall}{Precision+Recall})$$

7 $${\rm{P}}{\rm{r}}{\rm{e}}{\rm{c}}{\rm{i}}{\rm{s}}{\rm{i}}{\rm{o}}{\rm{n}}=\frac{TP}{TP+FP}$$

8 $${\rm{R}}{\rm{e}}{\rm{c}}{\rm{a}}{\rm{l}}{\rm{l}}=\frac{TP}{TP+FN}$$

where TN, TP, FP, FN refer to true negative, true positive, false positive and false negative respectively. *Ankistrodesmus* sp., which are similar in shape to short *Anabaena* segments, was added to the mixed suspension to demonstrate the algorithm’s capacity to differentiate cells of similar morphology. Confusion matrix analyses were conducted for individual pure culture to illustrate the performance of classification on the test data set when true values were known.

## Results and Discussion

### Contrast enhancement by fluorescence excitation

**Figure 2:**
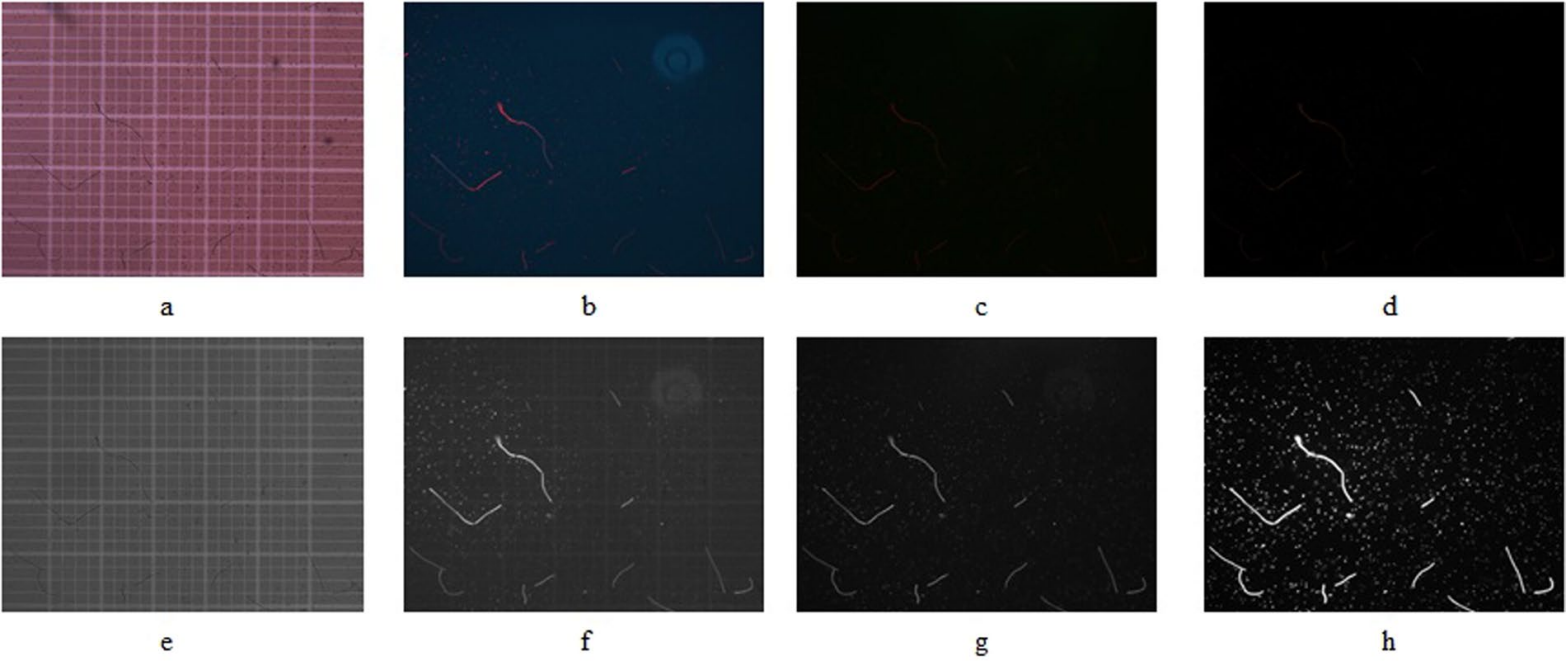
Representative images of a mixed suspension of *Microcystis aeruginosa* and *Anabaena flos-aquae* exposed to normal white light and lights at different excitation wavelengths. Note: Images (**a**,**b**,**c** and **d)** correspond to the RBG images exposed to normal white light, 365 nm, 450 nm and 546 nm; Images (**e,f,g** and **h**) correspond to mono images exposed to normal white light, 365 nm, 450 nm and 546 nm respectively; all figures refers to the same field view for demonstration purpose herein.

**Figure 3:**
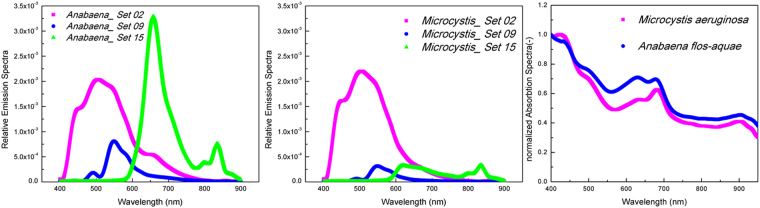
Fluorescence emission signals (**a** and **b**) and normalized absorption spectra (**c**) for *Anabaena flos-aquae* and *Microcystis aeruginosa* cells, respectively.

One of the major practical challenges for automated enumeration of cyanobacteria is the low and insufficient contrast between target cells and the surrounding background matrix (Fig. 2a and e). To overcome this technical barrier, specific wavelengths of light were utilized to excite natural photosynthetic pigments (i.e. chlorophyll-a and phycocyanin) present in the cells, causing them to fluoresce and thus achieving contrast enhancement. Figure 2b,c,d,f,g and h are representative images of a mixed suspension of *Microcystis aeruginosa* and *Anabaena flos-aquae* cells exposed to normal white light at different excitation wavelengths, specifically 365, 450–490, and 546 nm. When using the standard enumeration method^11^ in which cells in a hemocytometer are exposed to white light, it is difficult to enumerate using an automated algorithm^11,25,26^. Comparatively, when light at each of the selected wavelengths was used to excite the naturally fluorescent pigments within the cells, contrast was enhanced. This observation is in agreement with previous reports^26,30,45^. The spectrum of emission and absorption of the two cyanobacteria used were measured and are presented in Fig. 3. In the latter part of this paper, only the mono color images were used for further classification. While autofluorescence was used for contrast enhancement, the cell fluorescence/absorption information at different wavelengths was not used for quantification or discernment. Only morphological features were used for classification in the present work.

Zeiss filter set 2 (excitation wavelength of 365 nm) was found to enhance contrast the best with the strongest fluorescence emission signals for both *Anabaena* and *Microcystis* (Fig. 3a and b). For *Microcystis*, filter sets 9 (450–490 nm) and 15 (546 nm) had 8% and 18% of the power output of filter set 2, respectfully. In the case of *Anabaena*, filter sets 9 and 15 had 21% and 90% of the power output of filter set 2, respectively. These values match the relative output intensities of the Mercury Arc lamp light source in the microscope system used. Normalized absorption spectra data for the measured peak values of the two cyanobacteria within the 400–1000 nm range are represented in Fig. 3c. These absorption spectra were found to be similar, but still uniquely different, especially at wavelengths <680 nm. These observed emission and absorption spectrum signatures are consistent with the observed images suggesting different composition or amount of fluorescent pigments inside the cells of each of the cyanobacterial species studied. It should be emphasized that the cyanobacterial cells were all imaged within 1 hour of suspension preparation to ensure that the naturally fluorescent pigments were fully active. Additional experiments conducted using dry cells on quartz slides have shown that contrast enhancement stayed relatively strong up to 24 hours of dry preservation (data not shown). Based on these results, the images acquired using filter set 2 (excitation wavelength of 365 nm) were selected for further cell enumeration and biovolume estimation throughout this study.

### Model calibration

The model calibration process was the most critical step in the development of the proposed method. It involved quantitative evaluation of the cellular characteristics of the cyanobacteria, including dimensions and morphological features. It delivered the baseline statistics needed for cell and bio-volume enumeration. Various sonication periods for breaking cell aggregates and long filaments into shorter filaments or individual cell units prior to enumeration were evaluated. In general, as sonication period increased, the *Anabaena* filaments became shorter and the *Microcystis* cells less aggregated, as would be expected. Based on a qualitative assessment by microscopic examination, suspensions subjected to 2 minutes of sonication yielded the highest individual counts. This treatment time was thus selected for further use. The estimated 2 dimensional areas (x, y) of *Microcystis* and *Anabaena* cells were 24.6 ± 3.4 μm^2^ and 26.8 ± 2.5 μm^2^ respectively; and these are in agreement with commonly reported values^46^; thus, demonstrating the accuracy and efficiency of the proposed approach.

### Quantification of *Microcystis* and *Anabaena* concentration

**Figure 4:**
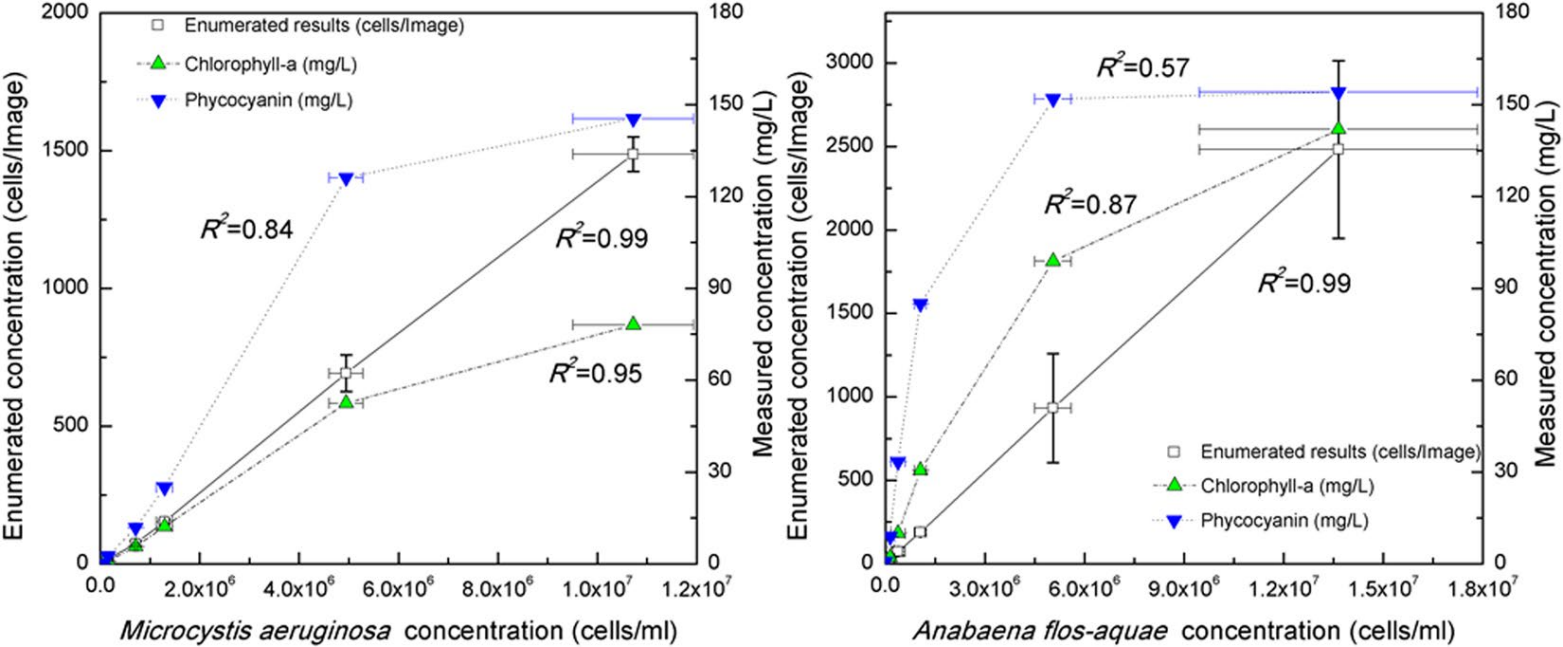
Quantitative comparison between *Microcysitis (***a***)* and *Anabaena* (**b**) cell concentrations (cells/ml) in PBS solution when obtained by the developed method and when indirectly estimated from chlorophyll–a and phycocyanin (mg/L) measurements using fluorometric probes. Error bars represent the standard derivations from multiple measurements.

Figure 4 shows the results obtained by manual cell enumeration using a hemocytometer according to Standard Methods (AWWA 10200 F) as a reference (abscissa) plotted against the values obtained for the same suspension (ordinate) using a) the proposed method and images from the same cell culture contained in the hemocytometer, and b) an indirect quantitative measurement of photosynthetic pigments (i.e. chlorophyll -a and phycocyanin) using two fluorometric probes (Fig. 4a). Five different initial cell concentrations were used for this quantitative comparison and the error bars represent the standard deviations from these multiple measurements. Ideally, such comparison would yield a coefficient of determination (*R*^2^) of 1.0 and a zero intercept to indicate perfect correspondence between counts obtained with the hemocytometer and those automatically enumerated with the developed method.

The *Microcystis* cell enumeration results obtained using the developed automated method closely corresponded to the reference measurements for cells suspended in PBS (*R*^2^ = 0.99; Fig. 4a). The determined accuracy (0.92 ± 0.05) and the F1 score (0.96 ± 0.03) for *Microcystis* indicate good enumeration of *Microcystis* cell concentration. In contrast, the estimated cell numbers based on concentrations of chlorophyll-a and phycocyanin (in mg/L) measured using the fluorometric probe correlated poorly with the reference values. When the cell concentration was high (>10^6^ cells/ml), the fluorometric probe significantly underestimated the concentration of *Microcystis cells*. At lower concentration ranges (10^4^–10^5^ cells/ml) however, it provided better estimates. The normalized portion of measured concentrations (mg/L) of chlorophyll-a to phycocyanin (*C*_*Cho*−a_/*C*_*Phyco*_) was approximately constant (40–53%), except at the lowest concentration (i.e. 7.5 × 10^4^ cell/ml) (Fig. S-1). This general consistency was expected because the cell suspensions were dilutions of the same raw culture; thus, the proportion of chlorophyll-a to phycocyanin should be consistent. This quantitative comparison suggests that the developed two-phased model provided an accurate measurement of cell concentrations over a wider range than the fluorometric probe.

To further validate the developed method for enumeration/biovolume estimation, laboratory cultured *Anabaena* cells were suspended in PBS at five dilution levels. As before, comparisons were conducted between the reference (abscissa) counts and those obtained using the developed method, as well as the fluorometric probes (ordinate). As shown in Fig. 4b, good correlation was found between the hemocytometer counts and those obtained using the proposed method (*R*^2^ = 0.99). The determined accuracy (0.86 ± 0.07) and the F1 score (0.91 ± 0.05) for *Anabaena* suggested reliable enumeration. It should be noted that for *Anabaena* counts the specificity and F1 scores were lower and the variability was higher than those found for *Microcystis*, regardless of cell concentration.

Good linear relationships between the reference *Anabaena* concentrations and the measured concentrations of chlorophyll–a and phycocyanin from the fluorometric probes (*R*^2^ = 0.57 and 0.87 respectively) were not observed (Fig. 4b). Given that the *Anabaena* suspensions were dilutions of the same original culture, the ratio of chlorophyll–a to phycocyanin was expected to be relatively constant. However, in this case the relative ratio of observed concentrations of chlorophyll–a to those of phycocyanin proved to be cell concentration dependent (Fig. S-2). This highlighted the limitations of the quantitative measurements of cyanobacterial cells using fluorometric probes, and underscored the advantages and usefulness of the proposed method. It should be kept in mind that spectrophotometric analyses only measure fluorescent signals at specific wavelengths and concentration estimates are based on assumptions of linearity between cell numbers and fluorescence intensity within certain cell concentration ranges. When the cell concentrations are outside of this range, there is inadequate sensitivity for lower values and signal saturation/dampening for higher values. In contrast, such limitations are not relevant to the developed direct enumeration method since the cyanobacterial fluorescence signatures are only used for identification purposes and to enhance contrast (i.e., there is no need to convert fluorescence to a cell count). Notably, the developed method generally yielded smaller standard deviations than the other methods. Perhaps more importantly, the imaging-based nature of the proposed method inherently provides a lower limit of detection than use of a hemacytometer because larger sample volumes (i.e., enumeration areas) can be processed, whereas a hemacytometer has a small fixed volume and requires relatively high concentrations for statistically valid concentration estimates, as discussed above. Moreover, where appropriate, the imaging-based may also be combined with filtration techniques that concentrate microorganisms present at lower concentrations (e.g., cyanobacteria prior to bloom conditions, parasites, etc.). Of course, the specific limits of detection would be microorganism and equipment (i.e., processed sample volume, area enumerated, etc.) specific—a detailed study of required enumeration areas/sample volumes to achieve targeted limits of detection was beyond the scope of this proof-of-concept investigation.

### Cell separation

**Figure 5:**
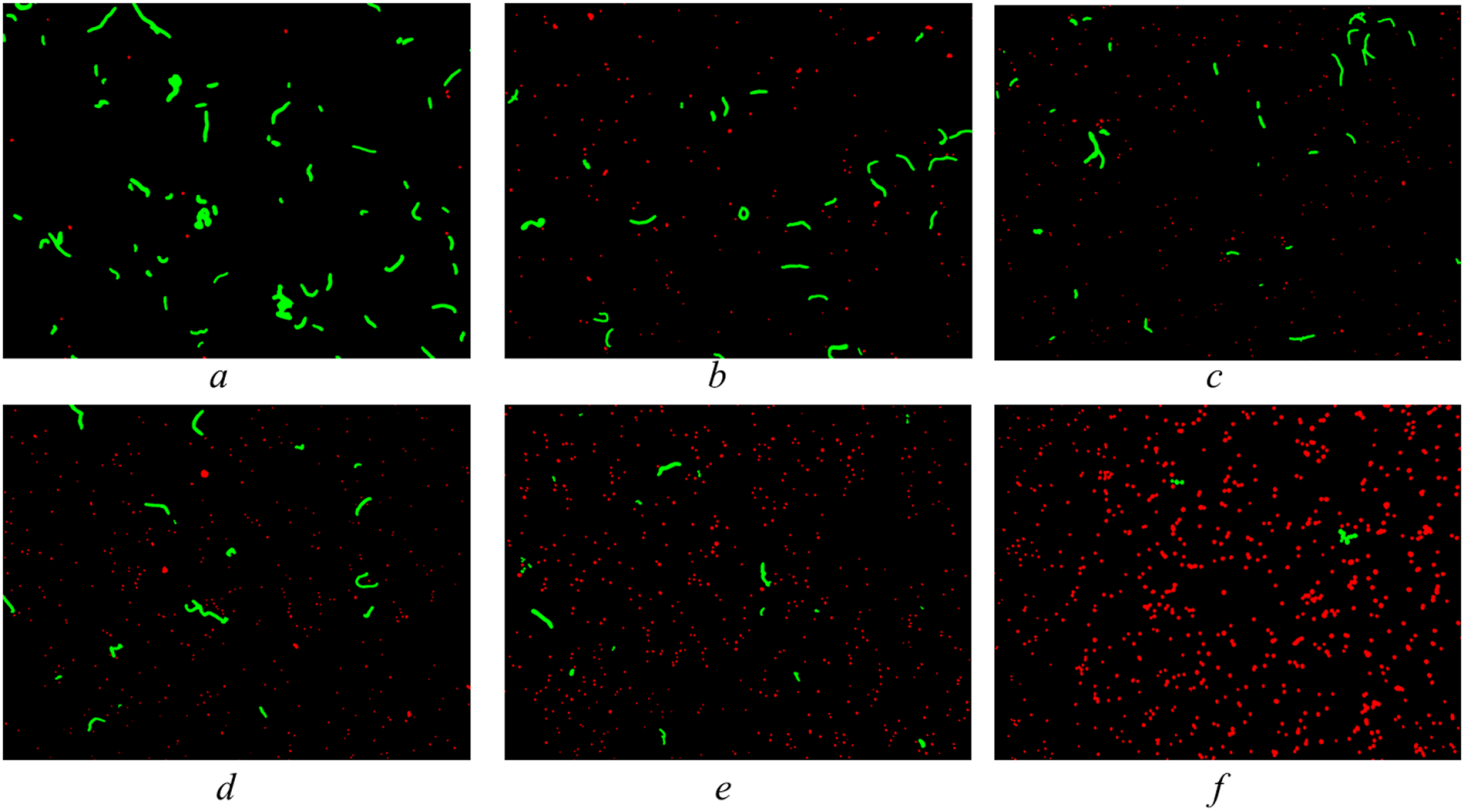
Representative processed mono fluorescent images of a mixed culture of *Microcysitis* (red) and *Anabaena* (Green) at different volumetric proportions, namely (**a**) 100, (**b**) 80, (**c**) 60, (**d**) 40, (**e**) 20 and (**f**) 0% of *Anabaena* cells (v/v).

**Figure 6:**
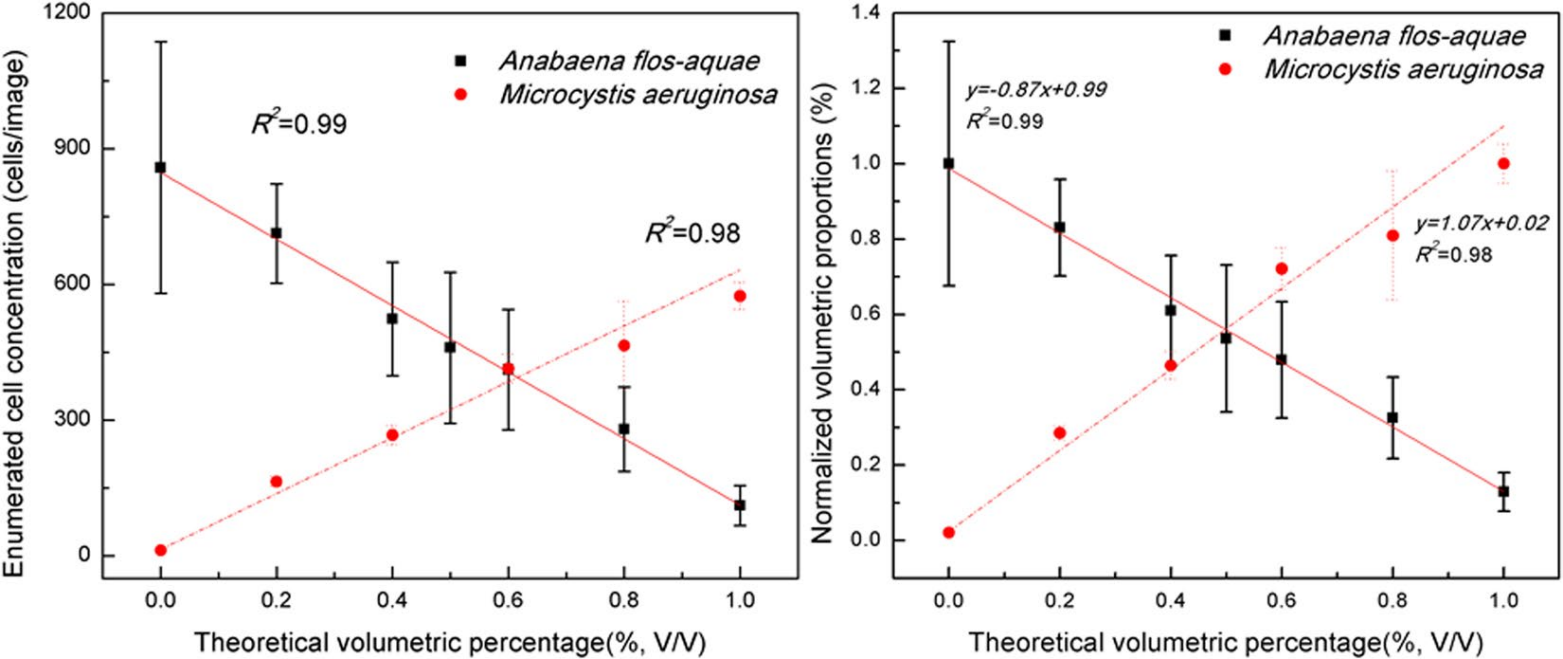
Enumeration results (cells/image) (**a**) and normalized volumetric proportions (%) (**b**) in a mixed culture of *Microcysitis* and *Anabaena* where error bars represent the standard deviations from the average of multiple measurements.

When several species of microorganisms are present (i.e. a mixed culture), cell separation constitutes another challenge to using automated enumeration. To validate the accuracy of the developed method, mixed cultures of *Anabaena flos-aquae* and *Microcystis aeruginosa* at different volumetric proportions were prepared and tested. Representative images of a mixed culture at different volumetric proportions are presented in Fig. 5. Clearly, in Fig. 5a to f, the number of *Anabaena* cells in the mixed suspension decreased while the number of *Microcystis* cells increased. Figure 6 describes the cell enumeration results and normalized volumetric proportions of both cyanobacterial species present in the mixed culture. The abscissa values are the calculated volumetric portions of the two species based on the dilution factors used (% in volume/volume) and the ordinate values are the enumeration results (Fig. 6a) or normalized volumetric proportions (Fig. 6b) using the developed method. The error bars represent the standard deviations for multiple measurements. The results obtained using the developed enumeration method showed good correlation (*R*^2^ = 0.98 for *Microcystis* and 0.98 for *Anabaena* counts respectively) (Fig. 6a); and the determined volumetric proportions of different species (Fig. 6b) were all in agreement with the theoretical values. In Fig. 6, the least squares linear regression of the cell concentration values using the automated method as compared to those obtained by manual count yield a slope of ±0.98, an intercept of 0.07 and 1.01, respectively). This analysis demonstrates that the developed two-phase method can be used for both accurate cell enumeration and differentiation between *Anabaena* from *Microcystis* cells in a mixed culture using the customized design screening criteria.

### Method validation using a real water matrix

**Figure 7:**
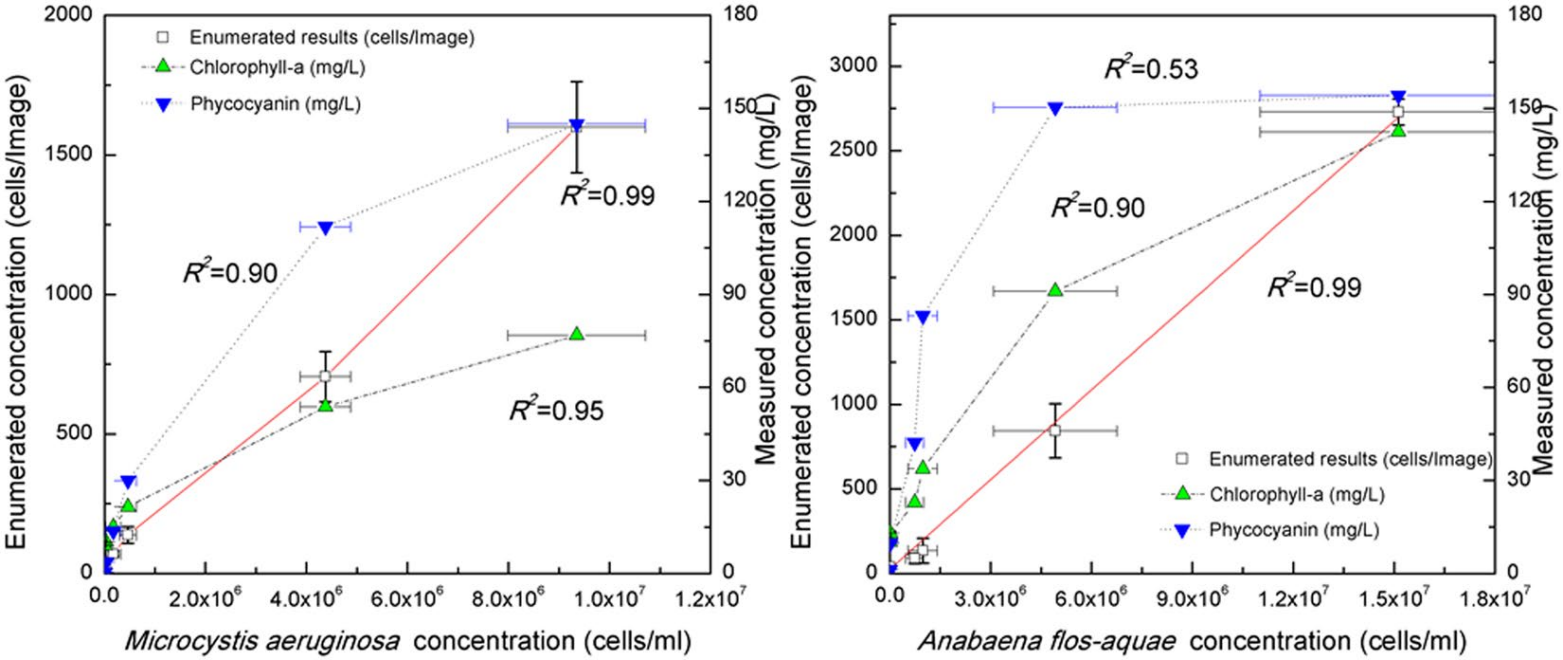
Quantitative comparison between *Microcysitis* (**a**) and *Anabaena* (**b**) cells concentrations (cells/ml) in lake water when obtained by the developed method (per image) and when indirectly estimated from chlorophyll–a and phycocyanin (mg/L) measurements using fluorometric probes. Error bars represent the standard derivations from multiple measurements.

Suspensions of *Microcystis* and *Anabaena* were diluted using untreated Lake Ontario water without any cyanobacterial cells (as confirmed by microscopic observation). Similar to what was observed with the PBS suspension, results obtained with the developed method presented a strong linear correlation to the manual enumeration results for both cyanobacteria species (Fig. 7). Compared to the results obtained using PBS, the enumeration results for cells suspended in lake water had higher variability, lower accuracy, and lower F1 score. As shown in Fig. S-3 for *Microcystis*, the relative proportion of chlorophyll–a over phycocyanin (mg/L) were significantly higher (260%) at the lower cell concentration range than those obtained at higher cell concentrations. As the concentration of *Microcystis* increased, the determined ratio gradually leveled off until it reached levels similar to those observed in the PBS suspension (~45%).

For *Anabaena*, the measured ratios of chlorophyll–a over phycocyanin achieved highest values (~140%) at low cell concentrations (5 × 10^4^ cells/ml) (Fig. S-4). When the concentration of *Anabaena* increased, the ratio of chlorophyll–a over phycocyanin became similar to the values found in the PBS suspensions. These observations are also evident in the evaluation of accuracy and in the F1 score (which also is a measure of accuracy). For both cyanobacteria, specificity and the F1 score were lower in lake water than obtained using the PBS matix, especially at lower cell concentrations. This is because un-wanted fluorescent objects (i.e. debris, clay particles and microorganisms) with various shapes and morphology present in natural water may have interfered with the quality of the collected images. This resulted in lower coefficients of determination and higher variability in cell counts as compared to those obtained in cleaner artificial water suspensions. In contrast, the newly developed enumeration method consistently provided accurate measurements of *Microcystis* and *Anabaena* numbers across the studied cell concentration ranges for both water matrices used. Further research is needed to: (1) compare experimental data obtained using the new method and those from other currently available image analysis-based enumeration techniques; and (2) develop the proposed method to distinguish between numerous cyanobacteria species and other biotic and abiotic suspended particles, as well as cells during various stages of their life cycle. A broader range of water matrices (e.g., higher turbidities) should also be evaluated.

### Differentiation of cells with relatively similar morphologies

**Figure 8:**
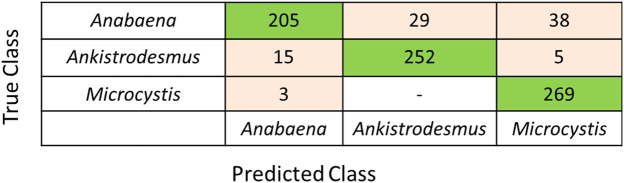
The confusion matrix tested on individual cells.

Cells of the green algae *Ankistrodesmus* cells are long and needle- or spindle-shaped, or sometimes curved or slightly crescent-shaped. While not identical, they can be similar in morphology to the shorter filaments of *Anabena*. Thus, they were added to the mixed cultures of *Microcystis* and *Anabaena* cells to investigate differentiation of different cell types with relatively similar morphologies. When only the two cyanobacteria were enumerated, the algorithm using three morphological features with a simple threshold classifier offered an effective and rapid method for classification and enumeration. To evaluate the mixed culture containing cells with relatively similar morphologies, a more complex classifier was used. Using the dataset of 816 images each consisting of either a *Microcystis*, *Anabaena*, or *Ankistrodesmus* cells, an exploration of different machine learning algorithms was first completed to identify the most effective method for identification. While auto-fluorescence was used for contrast enhancement, only morphological features were used for classification. It was found that a Support Vector Machine (SVM) with a quadratic kernel had the highest performance of 89.2% accuracy when classifying using five-fold cross-validation. The corresponding confusion matrix of this classification can be seen in Fig. 8.

**Figure 9:**
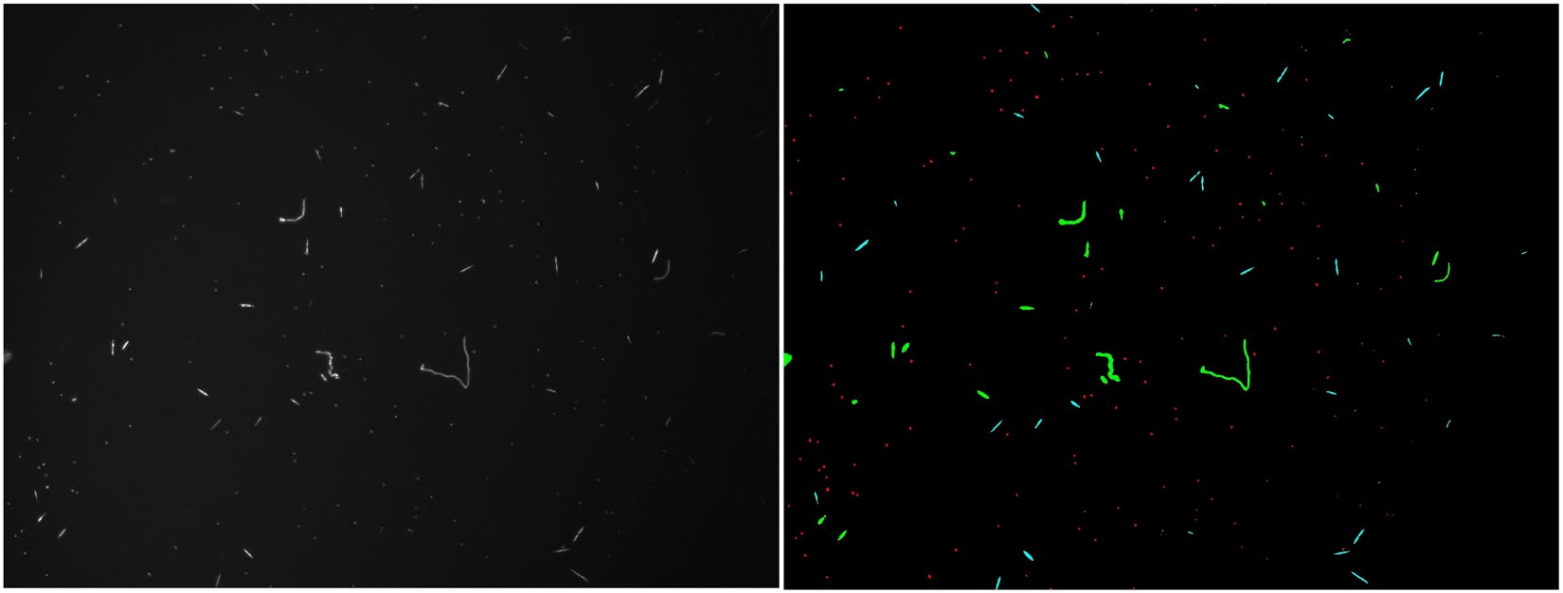
Raw (**a**) and processed (**b**) images of a mixed suspension of Microcystis (blue), Anabaena (red) and Ankistrodesmus (green), respectively.

The confusion matrix (Fig. 8) indicates that 15 *Ankistrodesmus* cells were mis-labeled as *Anabaena* in the processed images. This was due to the morphological similarities between small segments of *Anabaena* and cells of *Ankistrodesmus*. The cells of *Microcystis* were properly identified and distinguished as compared to *Anabaena* and *Ankistrodesmus*. As would be expected, the largest error of mis-identification resulted from the morphological similarities between *Anabaena* and *Ankistrodesmus*, especially when the segments of *Anabaena* were short. Having trained this classifier, an un-seen image with mixed cells was recorded (Fig. 9a) and analyzed (Fig. 9b) using the SVM classifier to automatically separate the three cell types; the latter image contains *Microcystis* (Blue), *Anabaena* (Red) and *Ankistrodesmus* (Green). The individual cells were classified based on the morphological attributes listed in Table 2 and correspond to the results presented in the confusion matrix in Fig. 8.

## Conclusions

Thus, the work presented herein demonstrates the proof-of-concept that contemporary image analysis and processing technology has advanced to a level that enables good identification and enumeration of cyanbacteria and other algae in real (untreated) water matrices in a manner that is (1) rapid and automated without requiring additional expensive equipment beyond that which is commonly available for microbiological analyses in typical water quality laboratories and (2) at least as accurate as currently available methods (e.g., hemacytometer, fluorometry), but with greater sensitivity (i.e., lower detection limit). Here, seven morphological features and a standard machine learning classifier such as a SVM in combination with additional tools such as fluorescence and absorption spectra were used.

When compared with the indirect or surrogate measurements based on pigment fluorescence estimation, the new method provides better accuracy at both low and high *Microcystis* cells concentrations,. For filamentous *Anabaena*, the proposed method (calibration-pre-treatment-contrast enhancement-enumeration process) provides better cell concentration estimation in the water matrices tested, as compared to the commonly used indirect pigment fluorescence measurement using fluorometric probes. Furthermore, the probe can not only accurately estimate cell concentrations, but also effectively distinguish between cells of *Anabaena flos-aquae* and *Microcystis aeruginosa* in a mixed culture. Indirect measurements only measure fluorescent intensity in response to differences in surrogate chemical(s), but cannot distinguish target cells from other cells containing chlorophyll-a or phycocianin or from other fluorescent particles present in natural water thus making it difficult to quantify the significance of false positive/negative results. Significant time and resources can be saved by using this type of method as compared to other cell enumeration methods in use for routine water monitoring. Notably, the concurrent analysis of *Microcystis*, *Anabaena*, and *Ankistrodesmus* demonstrated that imaging-driven machine learning approaches can be used to accurately differentiate between microorganisms with relatively similar morphologies. This work underscores the promise of these techniques and the need for their further investigation to develop reasonably low cost, rapid screening tools for evaluating water quality and public health risk.

## Electronic supplementary material

Supplementary information

## References

[1] Seckbach, J. *Algae and cyanobacteria in extreme environments* (Springer, 2007).

[2] Kulasooriya, S. Cyanobacteria: pioneers of planet earth. *Ceylon J. Sci. (Biol. Sci.)* **40** (2012).

[3] Havens, K. E. *Cyanobacterial harmful algal blooms: state of the science and research needs* 733–747 (Springer, 2008). 18561363

[4] Heil CA, Glibert PM, Fan C (2005). Prorocentrum minimum (Pavillard) Schiller: a review of a harmful algal bloom species of growing worldwide importance. Harmful Algae.

[5] Haag, A. L. Algae bloom again. *Nature* **447** (2007). 10.1038/447520a 17538590

[6] Chorus, E. I. & Bartram, J. Toxic cyanobacteria in water: a guide to their public health consequences, monitoring and management. (WHO, 1999).

[7] World Health Organization. Edition, Fourth. “Guidelines for drinking-water quality”. WHO chronicle 38.4: 104–8 (2011).

[8] Health Canada. Cyanobacterial Toxins in Drinking Water. http://www.healthycanadians.gc.ca/health-system-systeme-sante/consultations/cyanobacteria-cyanobacterie/alt/cyanobacteria-cyanobacterie-eng.pdf (Visited 2018.01) (2016).

[9] National Health and Medical Research Council, Australian Government. “Guidelines for Managing Risks in Recreational Water.” Canberra: Australian Government (Visited 2018.01) (2008).

[10] New Zealand guidelines. New Zealand Guidelines for Cyanobacteria in Recreational Fresh Waters. Interim Guidelines. http://www.mfe.govt.nz/publications/fresh-water-environmental-reporting/guidelines-cyanobacteria (Visited 2018.01) (2009).

[11] American Public Health Association. Standard methods for the examination of water and wastewater. Vol. 2. American Public Health Association. (Visited 2018.01) (1994).

[12] World Health Organization. Guidelines for safe recreational water environments: Coastal and fresh waters. Vol. 1. World Health Organization, (Visited 2018.01) (2003).

[13] Health Canada. Guidelines for Canadian Recreational Water Quality. https://www.canada.ca/en/health-canada/services/publications/healthy-living/guidelines-canadian-recreational-water-quality-third-edition.html (Visited 2018.01) (2012).

[14] Newcombe, G. ed. International guidance manual for the management of toxic cyanobacteria. Global Water Research Coalition, (Visited 2018.01) (2009).

[15] United States Environmental Protection Agency, Nutrient Pollution Policy and Data https://www.epa.gov/nutrient-policy-data. (Visited 2018.01).

[16] Box J (1981). Enumeration of cell concentrations in suspensions of colonial freshwater microalgae, with particular reference to Microcystis aeruginosa. British Phycological Journal.

[17] Federation, Water Environmental, and American Public Health Association. “Standard methods for the examination of water and wastewater.” American Public Health Association (APHA): Washington, DC, USA (2005).

[18] Marie D, Simon N, Vaulot D (2005). Phytoplankton cell counting by flow cytometry. Algal culturing techniques.

[19] Vrieling EG, Anderson DM (1996). Immunofluorescence in phytoplankton research: applications and potential. J. Phycol..

[20] Bolch CJ (2001). PCR protocols for genetic identification of dinoflagellates directly from single cysts and plankton cells. Phycologia.

[21] Popels LC (2003). The use of quantitative polymerase chain reaction for the detection and enumeration of the harmful alga Aureococcus anophagefferens in environmental samples along the United States East Coast. Limnology and Oceanography: methods.

[22] Al-Tebrineh J, Pearson LA, Yasar SA, Neilan BA (2012). A multiplex qPCR targeting hepato-and neurotoxigenic cyanobacteria of global significance. Harmful Algae.

[23] Scholin C, Buck K, Britschgi T, Cangelosi G, Chavez F (1996). Identification of Pseudo-nitzschia australis (Bacillariophyceae) using rRNA-targeted probes in whole cell and sandwich hybridization formats. Phycologia.

[24] Glöckner FO (1996). An *in situ* hybridization protocol for detection and identification of planktonic bacteria. Syst. Appl. Microbiol..

[25] He X (2016). Toxic cyanobacteria and drinking water: Impacts, detection, and treatment. Harmful Algae.

[26] Zamyadi, A., Choo, F., Newcombe, G., Stuetz, R. & Henderson, R. K. A review of monitoring technologies for real-time management of cyanobacteria: Recent advances and future direction. *TrAC Trends in Analytical Chemistry* **85**, Part A, 83–96 (2016).

[27] Orozco J, Medlin LK (2013). Review: advances in electrochemical genosensors-based methods for monitoring blooms of toxic algae. Environmental Science and Pollution Research.

[28] McQuaid N, Zamyadi A, Prévost M, Bird D, Dorner S (2011). Use of *in vivo* phycocyanin fluorescence to monitor potential microcystin-producing cyanobacterial biovolume in a drinking water source. Journal of Environmental Monitoring.

[29] Lefèvre F, Juneau P, Izquierdo R (2015). Integration of fluorescence sensors using organic optoelectronic components for microfluidic platform. Sensors Actuators B: Chem..

[30] Lefevre F (2012). Algal fluorescence sensor integrated into a microfluidic chip for water pollutant detection. Lab on a Chip.

[31] Juang, Y. & Chang, J. Applications of microfluidics in microalgae biotechnology: A review. *Biotechnology journal* (2016). 10.1002/biot.201500278 26807667

[32] Gray A, Young D, Martin N, Glasbey C (2002). Cell identification and sizing using digital image analysis for estimation of cell biomass in High Rate Algal Ponds. J. Appl. Phycol..

[33] Sezgin M (2004). Survey over image thresholding techniques and quantitative performance evaluation. Journal of Electronic imaging.

[34] Walker RF, Kumagai M (2000). Image analysis as a tool for quantitative phycology: a computational approach to cyanobacterial taxa identification. Limnology.

[35] Kass M, Witkin A, Terzopoulos D (1988). Snakes: Active contour models. International journal of computer vision.

[36] Zhang, Y. J. A review of recent evaluation methods for image segmentation (*Signal Processing and its Applications, Sixth International, Symposium on*. 2001 Ser. 1, IEEE, 2001).

[37] Sieracki ME, Reichenbach SE, Webb KL (1989). Evaluation of automated threshold selection methods for accurately sizing microscopic fluorescent cells by image analysis. Appl. Environ. Microbiol..

[38] Jin, C., Mesqutia, M., Emelko, M. & Wong, A. Automated enumeration and size distribution analysis of Microcystis aeruginosa via fluorescence imaging. *Journal of Computational Vision and Imaging Systems* **2** (2016).

[39] Rippka R, Deruelles J, Waterbury JB, Herdman M, Stanier RY (1979). Generic assignments, strain histories and properties of pure cultures of cyanobacteria. Microbiology.

[40] Smith, W. L., & Chanley, M. H. *Culture of marine invertebrate animals*. (Plenum Press, 1975).

[41] Dixon, W. J., & Massey, F. J. Jr *Introduction to statistical analysi*s.(1957).

[42] Student. On the error of counting with a haemacytometer. *Biometrika*, 351–360 (1907).

[43] Otsu N (1975). A threshold selection method from gray-level histograms. Automatica.

[44] Gonzalez, R. C., & Wintz, P. *Digital image processing*. (Addison-Wesley publishing company, 1987).

[45] Henderson R (2015). Fluorescence: State-of-the-art monitoring for water treatment systems. Water: Journal of the Australian Water Association.

[46] John, D. M., Whitton, B. A., & Brook, A. J. *The freshwater algal flora of the British Isles: an identification guide to freshwater and terrestrial algae* (Vol. 1). (Cambridge University Press, 2002).

