# Supplementary material for: Quantification of cyanobacterial cells via a novel imaging-driven technique with an integrated fluorescence signature
Source: Sci Rep. 2018 Jun 13;8:9055. doi: 10.1038/s41598-018-27406-0 (PMC5998128; doi:10.1038/s41598-018-27406-0)
Supplement: Supplementary file 1 — Supplementary information [file 41598_2018_27406_MOESM1_ESM.pdf]

Appendix A. supplementary information

Quantification of cyanobacteria cells via a novel imaging-driven technique with an integrated fluorescence signature

Chao Jin[†*], *Maria M. F.Mesquita*[†], *Jason L. Deglint, Monica B. Emelko*[†] *and Alexander Wong*[‡]

[†]Department of Civil and Environmental Engineering, University of Waterloo, Waterloo, Ontario N2L 3G1, Canada.

[‡]Department of Systems Design Engineering, University of Waterloo, Waterloo, Ontario N2L 3G1, Canada.

*Correspondence to j3chao@uwaterloo.ca

Number of Pages:6

Number of Figures: 4

Number of Tables:1

Table 1 – Comparison of the most commonly used methods for cyanobacteria cell enumeration

Quantification	Advantages	Disadvantages
**Direct Methods**		
Microscopic identification and enumeration of cells with sample in counting chamber or on a filter using:<br><br>1. Bright field<br>2. Differential interference contrast (DIC)<br>3. Epifluorescence	• high resolution<br>• quantification and identification occur simultaneously<br>• provides information on potential toxicity of species present	• time-consuming<br>• tedious<br>• accuracy and reproducibility depend on analyst's experience and taxonomic expertise<br>• may involve human error
**Indirect Methods**		
Microscopy combined with image acquisition and quantitative commercial image analysis software	• less time consuming than microscopy alone<br>• adds an extra level of documentation<br>• eases the quantification of cyanobacterial biomass when the dominant species is filamentous Minimizes human error	• errors involved specially when analyzing cells with a complex three dimensional geometry<br>• non-target particles (debris, contaminants may cause overestimation
Biomass or biovolume determination	• provides information on relative toxin content	• involves estimation errors
Photosynthetic pigments analyses<br><br>(1) chlorophyll-a analysis<br>(2) phycocyanin analysis<br><br>Spectral imaging of intracellular pigment<br><br>*In-situ* or remote monitoring of pigment fluorescence	• less time consuming than microscopy<br>• sensitive<br>• little analyst's experience required	• may vary according to physiological state of cells<br>• in mixed populations it gives an overestimation of cyanobacterial biomass<br>• less specific and less precise than microscopy<br>• no cell identification<br>• in some scenarios may cause interference and/or false readings
Flow cytometry	• rapid<br>• can use larger sample sizes<br>• high statistical confidence<br>• allows discrimination and quantification of target cells in complex samples<br>• may be combined with staining, antibody labelling and molecular probes	• high cost equipment<br>• requires trained personnel<br>• no cell identification<br>• cells need to be isolated
Molecular methods<br><br>• quantitative real-time polymerase chain reaction (qPCR)<br><br>• molecular probes using sandwich hybridization (SHA)	• rapid<br>• sensitive<br>• precise	• variability in extraction efficiencies, presence of inhibitory compounds and the design of standards for target species often compromise accuracy and reproducibility<br>• provides no cell identification<br>• requires trained personnel<br>• still under development

Sources: WHO 1999[1], Coyne et al. 2005[2], Vuorio et al. 2007[3], Australian-GWRC (2009)[4], Orozco and Medlin 2013[5], Peniuk et al. 2016[6], Health Canada 2016[7].

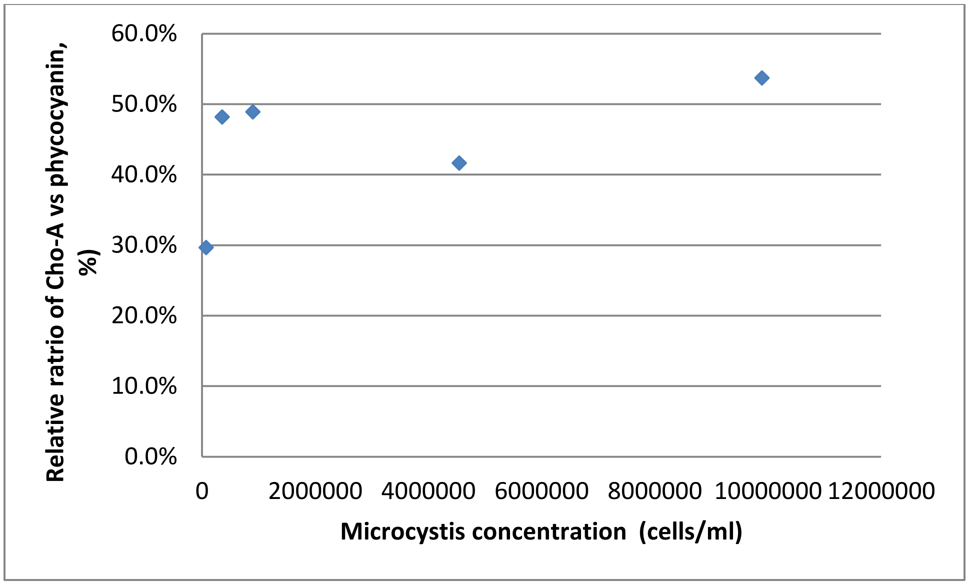

Figure S-1. Ratio of measured concentrations (mg/L) of chlorophyll-a over phycocyanin ($C_{Cho\text{-}a}/C_{Phyco}$) for *Microcystis* cells suspended in PBS

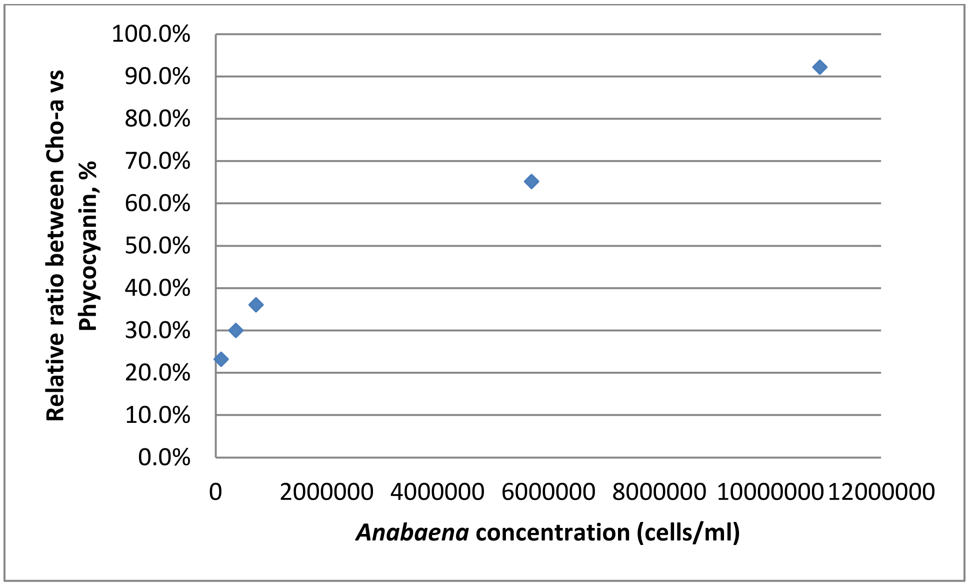

Figure S-2. Ratio of measured concentrations (mg/L) of chlorophyll-a over phycocyanin ($C_{Cho\text{-}a}/C_{Phyco}$) for *Anabaena* **cells suspended in PBS**

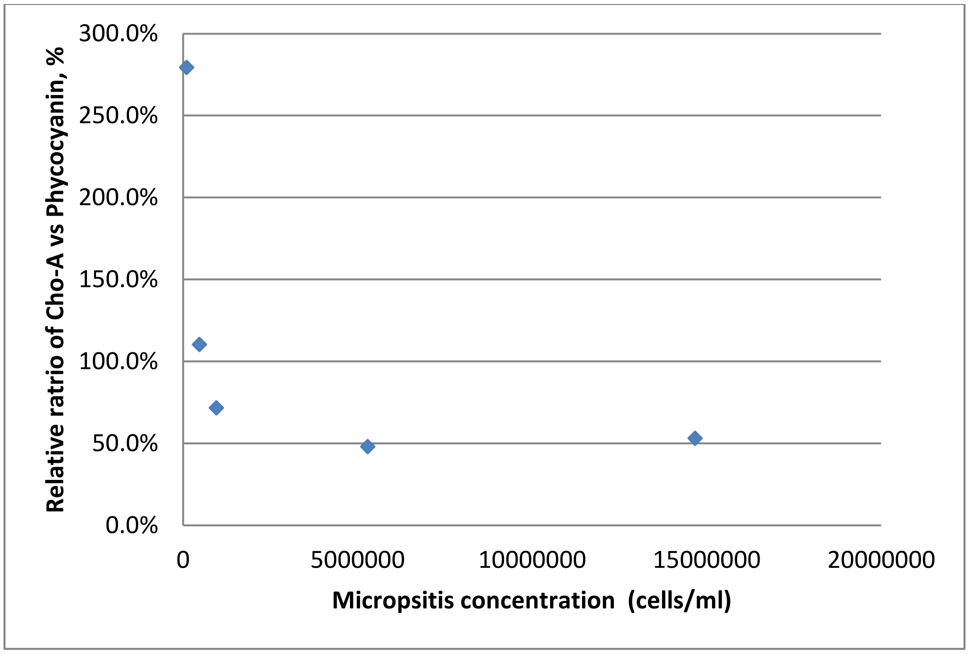

Figure S-3. Ratio of measured concentrations (mg/L) of chlorophyll-a over phycocyanin ($C_{Cho\text{-}a}/C_{Phyco}$) for *Microcystis* cells suspended in lake water

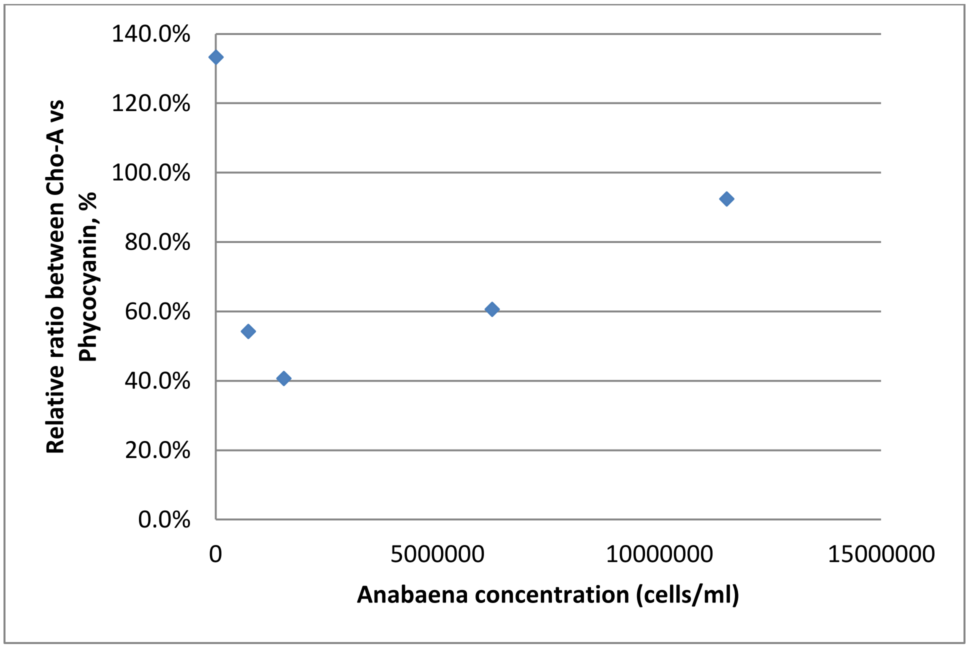

Figure S-4. The normalized portion of measured concentrations (mg/L) of chlorophyll-a over phycocyanin ($C_{Cho\text{-}a}/C_{Phyco}$) for *Anabaena* **cells suspended in lake water**

References

1. Chorus, E. I. & Bartram, J. Toxic cyanobacteria in water: a guide to their public health consequences, monitoring and management. (WHO,1999).

2. Coyne, K. J. *et al*. Improved quantitative real-time PCR assays for enumeration of harmful algal species in field samples using an exogenous DNA reference standard. *Limnology and Oceanography: Methods* **3**, 381-391 (2005).

3. Vuorio, K., Lepistö, L. & Holopainen, A. Intercalibrations of freshwater phytoplankton analyses. *Boreal Environ. Res.* **12** (2007).

4. Australian, G. International Guidance Manual for the Management of Toxic Cyanobacteria. *Australian, GWRC* http://www.waterra.com.au/cyanobacteria-manual/Chapter3.htm#Intro1 (2009).

5. Orozco, J. & Medlin, L. K. Review: advances in electrochemical genosensors-based methods for monitoring blooms of toxic algae. *Environmental Science and Pollution Research* **20**, 6838-6850 (2013).

6. Peniuk, G., Schnurr, P. & Allen, D. Identification and quantification of suspended algae and bacteria populations using flow cytometry: applications for algae biofuel and biochemical growth systems. *J. Appl. Phycol.* **28**, 95-104 (2016).

7. Health Canada. Cyanobacterial Toxins in Drinking Water . http://www.healthycanadians.gc.ca/health-system-systeme-sante/consultations/cyanobacteria-cyanobacterie/alt/cyanobacteria-cyanobacterie-eng.pdf (2016). (Visited 2018.01)
